# Branchial Na^+^:K^+^:2Cl^−^ cotransporter 1 and Na^+^/K^+^-ATPase α-subunit in a brackish water-type ionocyte of the euryhaline freshwater white-rimmed stingray, *Himantura signifer*

**DOI:** 10.3389/fphys.2013.00362

**Published:** 2013-12-10

**Authors:** Yuen K. Ip, Kum C. Hiong, Samuel Z. H. Wong, Biyun Ching, Xiu L. Chen, Melody M. L. Soh, You R. Chng, Jasmine L. Y. Ong, Jonathan M. Wilson, Shit F. Chew

**Affiliations:** ^1^Department of Biological Sciences, National University of SingaporeSingapore, Singapore; ^2^Ecofisiologia CIMARPorto, Portugal; ^3^Natural Sciences and Science Education, National Institute of Education, Nanyang Technological UniversitySingapore, Singapore

**Keywords:** elasmobranchs, gills, osmoregulation, rectal gland, salinity adaptation, urea, ureosmotic

## Abstract

*Himantura signifer* is a freshwater stingray which inhabits rivers in Southeast Asia. It can survive in brackish water but not seawater. In brackish water, it becomes partially ureosmotic, but how it maintains its plasma hypoionic to the external medium is enigmatic because of the lack of a rectal gland. Here, we report for the first time the expression of *Na^+^:K^+^:2Cl^−^ cotransporter 1* (*nkcc1*) in the gills of freshwater*H. signifer*, and its moderate up-regulation (~2-fold) in response to brackish water (salinity 20) acclimation. The absence of the Ste20-related proline-alanine-rich kinase and oxidation stress response kinase 1 interaction site from the N-terminus of *H. signifer* Nkcc1 suggested that it might not be effectively activated by stress kinases in response to salinity changes as in more euryhaline teleosts. The increased activity of Nkcc1 during salt excretion in brackish water would lead to an influx of Na^+^ into ionocytes, and the maintenance of intracellular Na^+^ homeostasis would need the cooperation of Na^+^/K^+^-ATPase (Nka). We demonstrated for the first time the expression of *nka*α*1*, *nka*α*2* and *nka*α*3* in the gills of *H. signifer*, and the up-regulation of the mRNA expression of *nka*α*3* and the overall protein abundance of Nkaα in response to acclimation to brackish water. Immunofluorescence microscopy revealed the presence of a sub-type of ionocyte, co-expressing Nkcc1 and Nkaα, near the base of the secondary lamellae in the gills of *H. signifer* acclimated to brackish water, but this type of ionocyte was absent from the gills of fish kept in fresh water. Hence, there could be a change in the function of the gills of *H. signifer* from salt absorption to salt excretion during brackish water acclimation in the absence of a functioning rectal gland.

## Introduction

The majority of elasmobranchs are marine, with few stenohaline freshwater species (Ballantyne and Robinson, 2010). Unlike marine teleosts which undergo hypoosmotic hypoionic regulation, marine elasmobranchs exhibit osmoconforming hypoionic regulation with their body fluid being isosmotic or slightly hyperosmotic to the environment (Yancey, 2001). Marine elasmobranchs actively regulate the ionic concentrations of their extracellular fluids lower (hypoionic) than those of the environment, with the osmotic difference balanced mainly by urea (Yancey, 2001; Ballantyne and Robinson, 2010). They maintain a functional ornithine-urea cycle in the liver (Anderson, 1980; Campbell and Anderson, 1991), and synthesize urea primarily for osmoregulatory purposes (Perlman and Goldstein, 1988; Ballantyne, 1997). As they accumulate high concentrations (300–500 mmol l^−1^) of urea inside their bodies, they are described as ureosmotic. Marine elasmobranchs uniquely possess rectal glands which secrete an almost pure NaCl solution of ~500 mmol l^−1^ and function in a capacity similar to the gills of marine teleosts for iono- and osmo-regulation (Pillans et al., 2005). Thus, gills of marine elasmobranchs serve only a minor role in ionoregulation despite being essential for acid–base balance (Ballantyne and Robinson, 2010).

Unlike teleosts, euryhalinity is an uncommon feature among elasmobranchs. Ballantyne and Robinson (2010) identified three stages in the colonization of fresh water by elasmobranchs. The first stage involves euryhaline marine species that can routinely enter and leave fresh water (e.g., bull shark, *Carcharhinus leucas*, and some populations of *Dasyatis sabina*). The second stage includes species that can live permanently in fresh water but are able to tolerate higher salinities. Examples of this group include some populations of *D. sabina* in the St. Johns River system of Florida (Johnson and Snelson, 1996), *Dasyatis garouaensis* from western Africa, and *Himantura signifer* from Southeast Asia. The third and final stage includes the species found only in fresh water that have no capacity to tolerate higher salinities. These are the stenohaline freshwater stingrays of the Family Potamotrygonidae that have been isolated in the Amazon basin of South America for about 65 million years (Lovejoy et al., 1998). Like freshwater teleosts, potamotrygonid stingrays gain water and lose ions in a hypoionic environment, and therefore their gills are involved in both salt absorption and acid-base regulation. Having lost the capacity to synthesize and accumulate urea (Thorson et al., 1967; Thorson, 1970), potamotrygonid stingrays have very low plasma urea concentrations (~1 mmol l^−1^), and cannot survive in waters of salinity 10 or above (Thorson, 1970; Tam et al., 2003).

The colonization of fresh water leads to a lower dependency of elasmobranchs on the salt-excreting function of the rectal gland and a greater dependence on the gills as a salt absorbing organ. For those euryhaline freshwater species that enter and leave fresh water routinely, the rectal gland is retained but does not function to excrete salt in fresh water. In fact, euryhaline species such as bull sharks (Thorson et al., 1978) and *Dasyatis guttata* (Thorson and Brooks, 1983) have smaller rectal glands even in seawater compared to their stenohaline congeners. Furthermore, the rectal gland of the euryhaline *D. sabina* in fresh water is 80% smaller than that of its marine counterpart (Piermarini and Evans, 1998). For stenohaline freshwater Potamotrygonids residing permanently in fresh water, their rectal glands are substantially reduced in size and non-functional (Thorson et al., 1978).

The white-edge freshwater whip ray, *H. signifer* (Compagno and Roberts) of Family: Dasyatidae inhabits rivers in Southeast Asia (Thailand, Indonesia and Papua New Guinea). In Indonesia, it can be found in the Batang Hari basin in Jambi, Sumatra, and is believed to thrive only in fresh water. However, it can travel freely along the river and may re-enter brackish/estuarine environments for reproduction (Otake et al., 2005). Recently, Wong et al. (2013) demonstrated that *H. signifer* might experience greater salinity-induced oxidative stress in fresh water than in brackish water, possibly related to its short history of fresh water invasion. In the laboratory, *H. signifer* can survive in fresh water (salinity 0.7) indefinitely or in brackish water (salinity 20) for at least two weeks (Tam et al., 2003; Ip et al., 2009). Unlike potamotrygonid stingrays, it possesses a functional urea cycle (Ip et al., 2003; Tam et al., 2003), despite having relatively low plasma urea concentrations (45–70 mmol l^−1^) in fresh water. During brackish water acclimation, it becomes partially ureosmotic, up-regulating urea synthesis and down-regulating urea permeability to increase tissue urea concentrations, albeit with a limited capacity (Tam et al., 2003; Ip et al., 2005; Chew et al., 2006). The plasma Na^+^ and Cl^−^ concentrations also increase, but they remain hypoionic to the ambient brackish water (Tam et al., 2003). Efforts have been made to identify and locate the rectal gland in numerous *H. signifer* specimens but to no avail (Chew and Ip, unpublished observation). Thus, how *H. signifer* maintains its plasma hypoionic to the external medium during brackish water acclimation remains an enigma.

Salt excretion occurs mainly in gills of marine teleosts. For those marine teleosts with a transepithelial electrical potential of 25–35 mV (blood side positive; Wright, 1991; Evans et al., 1999; Marshall, 2002), excess salt is excreted through ionocytes in the branchial epithelium, which involves the basolateral cotransport of Na^+^, K^+^, and Cl^−^ down the electrochemical gradient of Na^+^ provided by Na^+^/K^+^-ATPase (Nka), coupled with the apical exit of Cl^−^ via a protein channel and the paracellular extrusion of Na^+^. The key transporters involved are the basolateral Nka and Na^+^:K^+^:2Cl^−^ cotransporter 1 (Nkcc1), and the apical cystic fibrosis transmembrane conductance regulator Cl^−^ channel (Cftr; Hirose et al., 2003; Evans et al., 2005; Marshall and Grosell, 2006; Hwang and Lee, 2007; Evans, 2008; Hwang et al., 2011). By contrast, gills of elasmobranchs are not known to function as a salt excreting organ in a hyperionic environment. Nonetheless, for an euryhaline freshwater elasmobranch that lacks a functional rectal gland, its gills may serve an essential role in salt excretion in brackish water. Therefore, we hypothesized that during brackish water acclimation, *H. signifer* is capable of up-regulating some relevant branchial transporters to excrete excess salts through its gills.

Since no work has been done on the expression of *nkcc*/Nkcc in gills of elasmobranchs, the first objective of this study was to clone and sequence the cDNA of *nkcc1* from the gills of *H. signifer*, and to determine its mRNA expression in the gills of fish exposed to fresh water or brackish water (salinity 20) using quantitative real-time polymerase chain reaction (qPCR). The hypothesis tested was that gills of *H. signifer* expressed *nkcc1*, and exposure to brackish water would lead to an up-regulation of branchial *nkcc1* expression. The second objective was to elucidate the possible involvement of Nka in ionoregulation in the gills of *H. signifer* during brackish water (salinity 20) acclimation. Since NKA/Nka contains 2 major subunits, α and β, and the α-subunit contains all the functional sites and is responsible for the catalytic functioning of the enzyme, efforts were made to clone and sequence *nka* α-subunit isoforms from the gills of *H. signifer*. Four *NKA* α-subunits (α*1*, α*2*, α*3*, α*4*) have been identified in mammals (Blanco and Mercer, 1998). In the gill epithelia of euryhaline teleosts, isoforms of *nka*α specific to fresh water or seawater acclimation have been identified (Richards et al., 2003; Bystriansky et al., 2006, 2007; Ip et al., 2012). Thus, we hypothesized that multiple forms of *nka*α were expressed in the gills of *H. signifer*, and mRNA expression of certain forms of *nka*α would be selectively up-regulated for osmoregulatory purposes during brackish water acclimation. qPCR primers specific for each *nka*α isoform were designed to evaluate their mRNA expression in gills of fish exposed to fresh water or brackish water. In addition, Western blotting using a commercially available anti-NKA antibody was performed to test the hypothesis that brackish water acclimation would lead to an increase in the overall protein abundance of branchial Nkaα. Furthermore, efforts were made to identify the type of ionocyte, which co-expressed Nkcc and Nka in the basolateral membrane, using immunofluorescence microscopy. It was hoped that results obtained would shed light on the possible mechanisms involved in ionoregulation in the gills of the euryhaline freshwater *H. signifer* during brackish water acclimation.

In this report, two different types of abbreviations were adopted because the standard abbreviations of genes/proteins of fishes (http://zfin.org/cgi-bin/webdriver?MIval=aa-ZDB_home.apg) are different from those of human/non-human primates (http://www.genenames.org). Specifically, for fishes, gene symbols are italicized, all in lower case, and protein designations are the same as the gene symbol, but not italicized with the first letter in upper case. The advantage and appropriateness of using two types of abbreviations is that it would allow immediate interpretation of the affiliation between the abbreviated gene/protein and fish or human/non-human primates.

## Materials and methods

### Animals

Specimens of *H. signifer* were purchased from a local fish farm. Fish were kept in dechlorinated tap water (fresh water; pH 6.8–7.0) at 25°C in plastic tanks of appropriate sizes with aeration under a 12 h light: 12 h dark regime for at least 1 week before experiments. They were fed live shrimps daily. This study was approved by the Institutional Animal Care and Use Committee of the National University of Singapore (IACUC 021/10).

### Experimental conditions and collection of samples

Control fish (*N* = 4) were immersed in 25 volumes (v/w) of fresh water in plastic tanks, and they served as controls for the two experimental conditions: exposure to brackish water (salinity 20; pH 7.8) for 1 or 6 days. For exposure to salinity changes, fish (a total of *N* = 8) were transferred from fresh water (day 0) to waters of salinity 5 on day 1, salinity 10 on day 2, salinity 15 on day 3, salinity 20 on day 4 and kept in salinity 20 for 1 (*N* = 4) or 6 days (*N* = 4). Water was changed daily. Seawater was made from Red Sea salt (Houston, Texas, USA) and aerated for 24 h to obtain a stabilized pH of 8.2 before usage. Waters of different salinities were prepared by mixing seawater with an appropriate quantity of fresh water. Salinity was monitored using a YSI Model 30/10 FT salinometer (Yellow Springs Instrument Co. Inc, Ohio, USA). During salinity acclimation, stingrays were fed live shrimps on alternate days. Both control and experimental fish were killed by an overdose of neutralized 0.05% MS222, and their tissues quickly excised, frozen in liquid nitrogen and stored at −80^°^C until analysis.

### Total RNA extraction and cDNA synthesis

Total RNA was isolated from gill samples of *H. signifer* using TRI Reagent™ and purified using the Qiagen RNeasy Mini Kit (Qiagen GmbH, Hilden, Germany). The RNA was quantified spectrophotometrically using an Hellma TrayCell (Hellma GmbH & Co. KG, Müllheim, Germany) and its integrity checked electrophoretically to verify RNA by comparing the 18S and the 28S bands, which were visualized by a G:Box gel documentation system (Syngene, Cambridge, UK). Total RNA (1 μg) isolated was reverse transcribed into cDNA using RevertAid™ First Strand cDNA synthesis kit (Fermentas International Inc, Burlington, ON, Canada).

### Gene sequencing and cloning for isoform screening

Partial sequences of *nkcc* and *nka*α were obtained using PCR primers designed from the conserved regions of these genes (Table 1). PCR was carried out in Bio-Rad Peltier thermal cycler (Bio-Rad Laboratories, Hercules, CA, USA) using DreamTaq™ DNA polymerase (Fermentas International Inc.). The cycling conditions were 94°C (3 min), followed by 35 cycles of 94°C (30 s), 55°C (30 s), 72°C (2 min) and 1 cycle of final extension at 72°C (10 min). PCR products were electrophoresed in 1% agarose gel. Bands of the expected size were extracted from the gels using QIAquick® Gel Extraction Kit (Qiagen GmbH). Purified PCR products were subjected to cycle sequencing using BigDye® Terminator v3.1 Cycle Sequencing Kit (Applied Biosystems, Foster City, CA, USA) and purified by ethanol/sodium acetate precipitation. Purified products were automatically sequenced using the 3130XL Genetic Analyzer (Applied Biosystems).

**Table 1:** **Primer sequences for PCR, RACE-PCR and quantitative real-time PCR (qPCR)**.

**Gene**	**Primer type**		**(**5**′ → **3**′)**
*nkcc1*	PCR	Forward	GCWGCCACWGGYATT
		Reverse	GTSCCYTTSCCCTGHTTCTTCTG
	RACE-PCR	5′RACE	AGTTGTGCAGTTAGTCACTGTTCCAG
		3′RACE	ACGCAAGAACACAAGGATGAAGAAGATG
	qPCR	Forward	GTCGTCACCACCATCACAG
		Reverse	TCCAATAGCTCCTCCAAATTCAG
*nka*α	PCR	Forward	CACTTCATCCACATCATCAC
		Reverse	ATGGCAGGGAACCATGTC
*nka*α*1*	RACE-PCR	5′RACE	CCTGGAAGACTGCCCGGTTGCACA
		3′RACE	ACGCCATCATACTGAGATAGTCTTTGCT
	qPCR	Forward	AGTCTTCCAGGCAGGGCA
		Reverse	GCTCAACTGATCCACAACACAA
*nka*α*2*	RACE-PCR	5′RACE	TGGGTGAAGTCTTATCAAAGGCCGAA
		3′RACE	AAGAAGAGCAGCTAGACCAGATCCTG
	qPCR	Forward	GCCTTTGATAAGACTTCACCCAC
		Reverse	GCTGTCTCACGCTTGGAAAT
*nka*α*3*	RACE-PCR	5′RACE	TTTGTCGAAGGAAGCACCGGACTGG
		3′RACE	GAGCAGATTGACGACATCCTCCGG
	qPCR	Forward	GCGAGACAAGAACAAGAAGATT
		Reverse	GCCTCCTTCATCTCCTCATC

*Primers used for PCR, RACE and qPCR of Na^+^:K^+^:2Cl^−^ cotransporter 1 (nkcc1) and Na^+^/K^+^-ATPase (nka)* α*-subunit isoforms from gills of Himantura signifer.*

The PCR products obtained were subsequently ligated into pGEM-T easy vector (Promega Corporation, Madison, WI, USA). Ligation mixtures were then transformed into JM109 *Escherichia coli* competent cells. Standard Blue/ White screening was carried out on LB/ ampicillin/ Isopropyl β-D-1-thiogalactopyranoside (IPTG)/ bromo-chromo-iodolyl-galactopyranoside (X-gal) plates and all white colonies were selected. Colony-PCR was performed on all the selected white colonies. Colonies with insert of expected sizes were selected and grown overnight in LB/ampicillin broth in a shaking incubator (37°C, 250 rpm). Plasmid extraction was performed using AxyPrep Plasmid Miniprep Kit (Axygen Biosciences, Union City, CA, USA), in accordance to AxyPrep Plasmid Miniprep Spin Protocol (Axygen Biosciences). The concentration of recombinant plasmid DNAs was determined spectrophotometrically and sequenced. The partial sequences obtained were verified to be *nkcc1, nka*α*1*, *nka*α*2*, and *nka*α*3* from Genbank database.

### Rapid amplification of cDNA ends (race)-PCR

Total RNA (1 μg) was reverse transcribed into 5′-RACE-Ready cDNA and 3′RACE-Ready cDNA using SMARTer^TM^ RACE cDNA Amplification kit (Clontech Laboratories, Mountain View, CA, USA). RACE-PCR was performed using the Advantage®2 PCR kit (Clontech Laboratories) to generate the 5′ and 3′ cDNA fragments, using specific RACE-PCR primers (Table 1). RACE-PCR cycling conditions were 25 cycles of 94°C for 30 s, 65°C for 30 s and 72°C for 4 min. RACE-PCR products were separated using gel electrophoresis, purified and sequenced. Multiple sequencing was performed in both directions to obtain the full-length cDNA. Sequence assembly and analysis were performed using BioEdit 7.0.9 (Hall, 1999).

### Deduced amino acid sequence and phylogenetic analysis

The nucleotide sequences obtained were translated into amino acid sequences using ExPASy Proteomic server (Gasteiger et al., 2003). The potential phosphorylation, *O*-GlcNAcylation and *N-GlcNAcylation* sites were predicted using NetPhos 2.0 (Blom et al., 1999), YinOYang 1.2 (Gupta, 2001; Gupta and Brunak, 2003) and NetNglyc 1.0, respectively. The transmembrane regions (TMs) were predicted using MEMSAT3 & MEMSAT-SVM provided by PSIPRED protein structure prediction server (http://bioinf.cs.ucl.ac.uk/psipred/) (McGuffin et al., 2000).

The relationships between the translated amino acid sequences of Nkcc1 or Nkaα isoforms from *H. signifer* with other animals were analyzed using the neighbor-joining method (NEIGHBOUR) in PHYLIP phylogeny package (version 3.67), with the inclusion of 100 bootstraps (Felsentein, 1989). The phylogenetic tree was generated with CONSENSE using 50% majority rule and plotted using the TREEVIEW program version 1.6.6 (Page, 1996). Bootstrap values were indicated at the nodes of the tree branches. The Phylip analysis served two purposes: (1) to produce phenograms of amino acid sequence similarities to confirm the identities of the deduced amino acid sequences of Nkcc and Nkaα isoforms from the gills of *H. signifer*, besides using protein BLAST and comparing with other sequences based on ClustalX2 and Bioedit, and (2) to evaluate the relationships of those deduced amino acid sequences from *H. signifer* and those from other fishes (mainly teletosts) or tetrapods.

Complete amino acid sequences of Nkcc/NKCC from other animals (except those specified) were obtained from Genbank of UniProtKB/TrEMBL with the following accession numbers: *Anabas testudineus* Nkcc1a (AFK29496.1), *Anguilla anguilla* Nkcc1a (CAD31111.1), *A. anguilla* Nkcc1b (CAD31112.1), *Callorhinchus milii* Nkcc1 (BAN42614.1; 1055 amino acid residues), *C. milii* Nkcc2 (BAN42615.1; 862 amino acid residues), *Danio rerio* Nkcc1 (NP_001157126.1), *Dicentrarchus labrax* Nkcc1 (ABB84251.1), *Homo sapiens* NKCC1 (P55011.1), *H. sapiens* NKCC2 (NP_000329.2), *Monopterus albus* Nkcc1b (KC800686), *Mus musculus* NKCC1 (NP_033220.2), *M. musculus* NKCC2 (CAM17720.1), *Oreochromis mossambicus* Nkcc1a (AAR97731.1), *O. mossambicus* Nkcc1b (AAR97732.1), *O. mossambicus* Nkcc2 (AAR97733.1), *Paralichthys olivaceus* Nkcc (BAK74831.1), *Rattus norvegicus* NKCC1 (NP_113986.1), *R. norvegicus* NKCC2 (NP_001257547.1), *Sarotherodon melanotheron* Nkcc1 (ACY05529.1), *Squalus acanthias* Nkcc1 (AAB60617.1), *Takifugu obscurus* Nkcc2 (BAH20440.1), *Triakis scyllium* Nkcc2 (BAN42608.1; 312 amino acid residues), *Xenopus laevis* Nkcc1 (ABN05233.1), and *Strongylocentrotus purpuratus* Nkcc (NP_001106707.1; as an outgroup).

Complete amino acid sequences of Nkaα/NKAα from other animals were obtained from Genbank of UniProtKB/TrEMBL with the following accession numbers: *A. anguilla* Nkaα1 (Q92030), *Bufo marinus* NKAα1 (P30714), *Carassius auratus* Nkaα3 (BAB60722), *D. rerio* Nkaα2 (NP_571758), *Fundulus heteroclitus* Nkaα1 (AAL18002), *F. heteroclitus* Nkaα2 (AAL18003), *Gallus gallus* NKAα1 (NP_990852), *G. gallus* NKAα2 (NP_990807), *G. gallus* NKAα3 (NP_990806), *H. sapiens* NKAα1 (NP_000692), *H. sapiens* NKAα3 (NP_689509), *M. musculus* NKAα1 (AAH33435), *M. musculus* NKAα2 (AAH36127), *M. musculus* NKAα3 (NP_659170), *Oncorhynchus masou* Nkaα1a (BAJ13363), *O. masou* Nkaα1b (BAJ13362), *Oncorhynchus mykiss* Nkaα1a (NP_001117933), *O. mykiss* Nkaα1b (NP_001117932), *O. mykiss* Nkaα2 (NP_001117930), *O. mykiss* Nkaα3 (NP_001118102), *O. mossambicus* Nkaα1 (AAD11455), *O. mossambicus* Nkaα3 (AF109409_1), *R. norvegicus* NKAα1 (AAA41671), *Salmo salar* Nkaα1 (ACN10460), *S. melanotheron* Nkaα1 (ADB03120), *S. acanthias* Nkaα (CAG77578), *Torpedo californica* Nkaα (P05025.1), *Trematomus bernacchii* Nkaα3 (AAY30258), *X. laevis* Nkaα1 (NP_001084064), *X. laevis* Nkaα2 (NP_001083112.1), *X. laevis* Nkaα3 (NP_001080440.1), *Xenopus (Silurana) tropicalis* Nkaα1 (NP_989407), *X. tropicalis* Nkaα2 (NP_001096439.1), *X. tropicalis* Nkaα3 (NP_001120366.1) and *Saccoglossus kowalevskii* Nkaα1 (XP_002737354; as an outgroup).

### qPCR

Total RNA was isolated from gill samples of *H. signifer* using TRI Reagent™ protocol and purified using the Qiagen RNeasy Plus Mini Kit (Qiagen GmbH). Total RNA (1 μg) from the gill sample was reverse transcribed using random hexamer primers with RevertAid™ first strand cDNA synthesis kit (Fermentas International Inc.). qPCR was performed in triplicates using a StepOnePlus™ Real-Time PCR System (Life Technologies Corporation, Carlsbad, CA, USA). The mRNA expression of *nkcc1*, *nka*α*1*, *nka*α*2*, and *nka*α*3* in the gills of *H. signifer* were determined using specific qPCR primers (Table 1).

Since it was essential to compare the mRNA expression of *nkcc1* in the gills of *H. signifer* with those of other freshwater fishes, and compare the mRNA expression of *nka*α*1*, *nka*α*2*, and *nka*α*3* in the gills of *H. signifer*, the method of absolute quantification with reference to a standard curve was adopted in this study. Relative quantitation methods produce only fold-change data but do not allow the interpretation of which isoform being the predominant one expressed under a certain condition. Although absolute quantification provides more information, it is considered to be more labor-intensive than relative quantification. Absolute quantification is not commonly adopted because of the necessity to create reliable standards for quantification and include these standards in every PCR. Therefore, to determine the absolute quantity of transcripts of *nkcc1* and each of the 3 *nka*α in a qPCR reaction, efforts were made to produce a pure amplicon (standard) of a specific region of each of the 4 cDNAs, as defined by the specific qPCR primers, from the gills of *H. signifer* following the method of Gerwick et al. (2007). PCR was performed with a specific set of qPCR primers (Table 1) and cDNA as a template in a final volume of 25 μl with the following cycling conditions: initial denaturation 95°C for 3 min, followed by 35 cycles of 95°C for 30 s, 60°C for 30 s and 72°C for 30 s and 1 cycle of final extension of 72°C for 10 min. The PCR product was separated in a 2% agarose gel then excised and purified using FavorPrep™ Gel Purification Mini Kit (Favorgen Biotech Corp., Ping-Tung, Taiwan). The nucleotide fragment in the purified product was cloned using pGEM®-T Easy vector (Promega Corporation). The presence of the insert in the recombinant clones was confirmed by sequencing. The cloned circular plasmid was quantified using a spectrophotometer with a Hellma TrayCell.

The standard cDNA (template) was serially diluted (from 10^6^ to 10^2^ specific copies per 2 μl). The PCR reactions contained 5 μl of 2× Fast SYBR®Green Master Mix (Life Technologies Corporation), 0.3 μmol l^−1^ of forward and reverse primers each (Table 1) and 1 ng of sample cDNA or various quantities of standard in a total volume of 10 μl. Cycling conditions were 95°C for 20 s (1 cycle), followed by 40 cycles of 95°C for 3 s and 60°C for 30 s. Data (C_t_ values) were collected at each elongation step. A melt curve analysis was performed after each run by increasing the temperature from 60 to 95°C in 0.3°C increments to confirm the presence of a single product only. The PCR products obtained were also separated in a 2% agarose gel to verify the presence of a single band. A standard curve was obtained from plotting threshold cycle (C_t_) on the Y-axis and the natural log of concentration (copies μl^−1^) on the X-axis. The Ct slope, PCR efficiency, Y-intercept and correlation coefficient (*R*^2^) were calculated using the default setting of StepOne™ Software v2.1 (Life Technologies Corporation). Diluted standards were stored at −20^°^C. The PCR efficiencies for *nkcc1*, *nka*α*1*, *nka*α*2*, and *nka*α*3* were 101.6, 104.1, 97.1, and 101.7%, respectively. The quantity of transcript in a sample was determined from the linear regression line derived from the standard curve and expressed as copy number per ng cDNA.

For *nka*α, the specificity of each pair of qPCR primers was verified by PCR using three different plasmid clones containing fragments of *nka*α*1*, *nka*α*2*, and *nka*α*3* as templates. The identities of these plasmid clones had previously been verified through cloning and sequencing (see above). The specificity of each pair of primers was demonstrated by the presence of a single band using the plasmid clones of the targeted *nka*α isoform as the template and the absence of detectable band using the plasmid clones of the other two isoforms. Furthermore, for each pair of primers, the C_t_ value obtained using plasmid clones of the targeted *nka*α fell between 16 and 20, but no C_*t*_ values (i.e., undetermined) were obtained using the other two plasmid clones.

### SDS-page and western blotting

Gill filaments were homogenized three times in five volumes (w/v) of ice cold buffer containing 50 mmol l^−1^ Tris HCl (pH 7.4), 1 mmol l^−1^ EDTA, 150 mmol l^−1^ NaCl, 1 mmol l^−1^ NaF, 1 mmol l^−1^ Na_3_VO_4_, 1% NP-40, 1% sodium deoxycholate, 1 mmol l^−1^ PMSF, and 1x HALT™ protease inhibitor cocktail (Thermo Fisher Scientific Inc., Waltham, MA, USA) at 24,000 rpm for 20 s each with 10 s intervals using the Polytron PT 1300D homogenizer (Kinematica AG, Lucerne, Switzerland). The homogenate was centrifuged at 10,000 × *g* for 20 min at 4°C. The protein concentration in the supernatant obtained was determined according to the method of Bradford (1978) and adjusted to 5 μg μl^−1^ with Laemmli buffer (Laemmli, 1970). Samples were heated at 70°C for 15 min, and then kept at −80C until analysis.

Proteins were separated by SDS-PAGE (8% acrylamide for resolving gel, 4% acrylamide for stacking gel) under conditions as described by Laemmli (1970) using a vertical mini-slab apparatus (Bio-Rad Laboratories). Proteins were then electrophoretically transferred onto PVDF membranes using a transfer apparatus (Bio-Rad Laboratories). After transfer, membranes were blocked with 10% skim milk in TTBS (0.05% Tween 20 in Tris-buffered saline: 20 mmol l^−1^ Tris-HCl; 500 mmol l^−1^ NaCl, pH 7.6) for 1 h before being incubated overnight at 4°C with anti-NKA antibody (α5, 1:400 dilution). The α5 antibody was obtained from the Developmental Studies Hybridoma Bank maintained by the University of Iowa, Department of Biological Sciences, Iowa City, IA, USA. The α5 antibody was developed by Douglas M. Farmbrough (Johns Hopkins University, MD, USA) and is known to react comprehensively with Nka α-subunit isoforms in fish. The primary antibody was diluted in 1% bovine serum albumin in TTBS. The membranes were then incubated in goat anti-mouse horseradish peroxidase-conjugated secondary antibody (1:5000 dilution; Santa Cruz Biotechnology, CA, USA) for 1 h at room temperature. Bands were visualized by chemiluminescence (Western Lightning™, PerkinElmer Life Sciences, Boston, MA, USA) using X-ray film (Thermo Fisher Scientific) and were processed by a Kodak X-Omat 3000 RA processor (Kodak, Rochester, NY, USA). The films were scanned using CanonScan 4400F flat bed scanner in TIFF format at 300 dpi resolution. Densitometric quantification of band intensities were performed using ImageJ (version 1.40, NIH), calibrated with a 37 step reflection scanner scale (#R3705-1C; Stouffer Graphic Arts, South Bend, IN, USA). Results were presented as arbitrary densitometric units per mg protein.

Efforts were also made to determine the protein abundance of Nkcc1 using the T4 anti-NKCC antibody (Developmental Studies Hybridoma Bank, Iowa City, IA, USA) but to no avail. One possible explanation is that Nkcc expression was restricted to few specific cells in the gills of *H. signifer* as demonstrated by immunofluorescence microscopy.

### Immunofluorescence microscopy

Immunofluorescence microscopy was performed on gill filaments of the first and second gill arches from three different fish in fresh water (control) or acclimated to brackish water (salinity 20) for 6 days. Antigen retrieval was performed by treating deparaffinized sections with 0.05% citraconic anhydride (Namimatsu et al., 2005) and 1% sodium dodecyl sulfate solution. Sections were subsequently labeled using anti-NKCC/NCC mouse monoclonal antibody (T4; Developmental Studies Hybridoma Bank) and anti-NKA αRb1 rabbit polyclonal antibody. The T4 antibody developed by Christian Lytle (University of California Riverside, Riverside, CA) was obtained from the Developmental Studies Hybridoma Bank developed under the auspices of the National Institute of Child Health and Human Development and maintained by Department of Biological Sciences, The University of Iowa, Iowa City, IA. The anti-NKA αRb1 is a pan-specific antibody originally designed by Ura et al. (1996) for labeling Nka α-subunit isoforms which is widely used for fish species (Wilson, 2007). T4 and αRb1 were diluted 1:100 and 1:500, respectively, in blocking buffer (1% BSA in TPBS). Primary antibody incubations were performed at 37°C for 1 h. Secondary antibody incubations using goat anti-rabbit Alexa Fluor® 488 and goat anti-mouse Alexa Fluor® 568 (1:500 dilution for both secondary antibodies; Life Technologies Corporation) were carried out at 37° C for 1 h. After primary and secondary antibody incubations, sections were rinsed three times with TPBS and mounted. The corresponding differential interference contrast (DIC) image was also captured for tissue orientation. Sections were then viewed on an Olympus BX60 epifluorescence microscope (Olympus Corporation, Tokyo, Japan) and images captured using the Olympus DP73 digital camera (Olympus Corporation). Optimal exposure settings were predetermined and all images captured under these settings. Brightness and contrast of the plates were adjusted while maintaining the integrity of the data.

#### Statistical analysis

Results are presented as means ± standard errors of means (s.e.m.). Statistical analyses were performed using SPSS version 21 (IBM Corporation, Armonk, NY, USA). Homogeneity of variance was checked using Levene's Test. Differences between means were tested using Student's *t*-test (for Figure 10) or One-Way analysis of variance (ANOVA) followed by multiple comparisons of means by Dunnett's T3 (for Figure 7 with unequal variance) or by Tukey's test (Figures 3, 8 and 9 with equal variance). Differences were regarded as statistically significant at *P* < 0.05.

## Results

### Nucleotide sequences of *nkcc1*, and its deduced amino acid sequences

**Figure 1:**
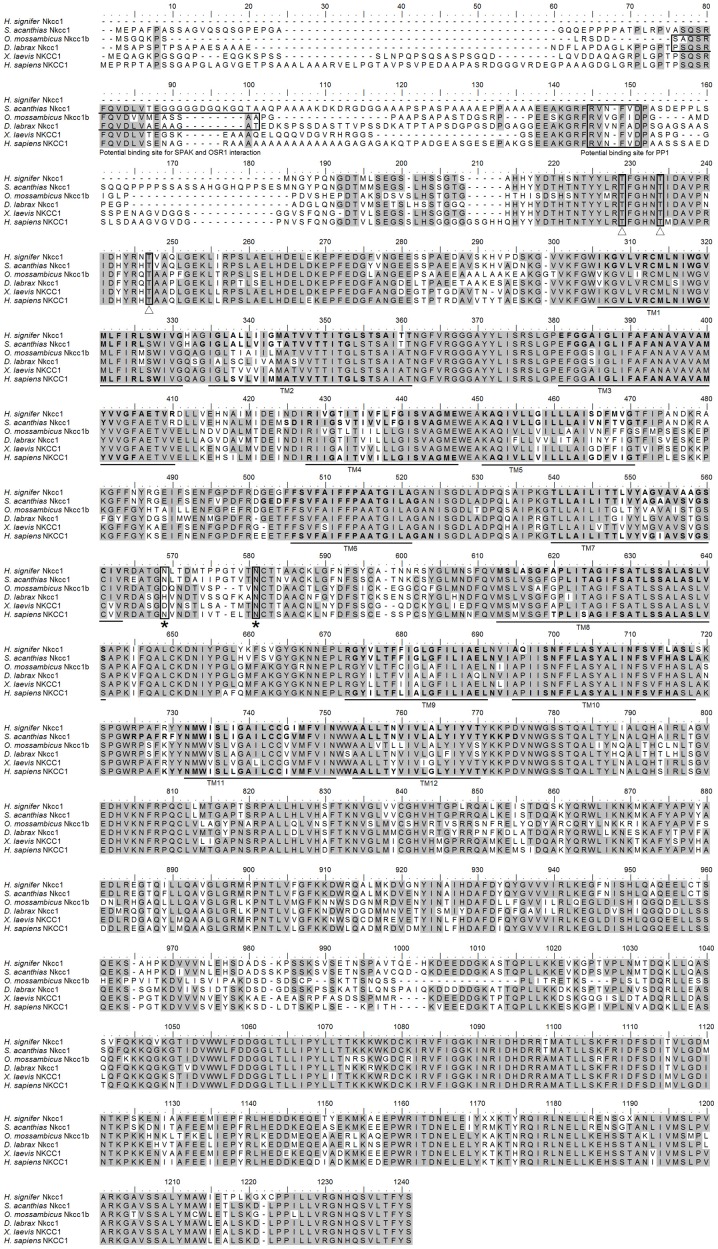
**Molecular characterization of Na^+^:K^+^:2Cl^−^ cotransporter 1 (Nkcc1) from the gills of *Himantura signifer*.** A multiple sequence alignment of the Nkcc1 from the gills of *H. signifer* with five other known Nkcc1/NKCC1 amino acid sequences from other animal species with Genbank accession numbers: *Squalus acanthias* (AAB60617.1), *Oreochromis mossambicus* (AAR97732.1), *Dicentrarchus labrax* (ABB84251.1), *Xenopus laevis* (ABN05233.1) and *Homo sapiens* (P55011.1). Shaded residues denote identical residues. The Ste20-related proline-alanine-rich-kinase (SPAK) and oxidation stress response kinase 1 (OSR1) interaction site and the protein phosphatase 1 (PP1) interaction site which are absent in *H. signifer* but present in other fishes are indicated with boxes. Predicted phosphorylation sites are indicated with boxes and open triangles while potential *N*-glycosylation sites are indicated with boxes and asterisks. The predicted transmembrane domains (TM) are underlined.

**Figure 2:**
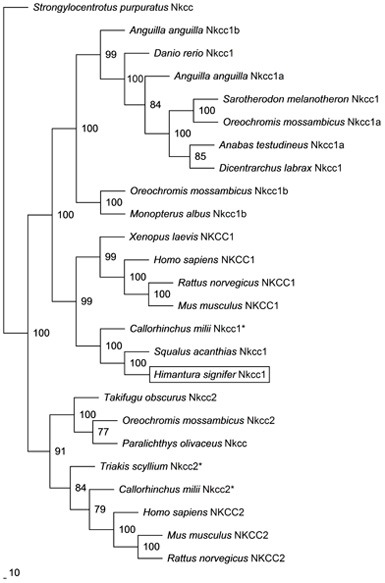
**Phylogenetic analysis of Na^+^:K^+^:2Cl^−^ cotransporter 1 (Nkcc1) from the gills of *Himantura signifer*.** A phylogenetic tree to illustrate the relationship between Nkcc1 from the gills of *H. signifer* and Nkcc/NKCC of selected vertebrate species. Numbers presented at each branch point represent bootstrap values from 100 replicates. *Strongylocentrotus purpurtus* Nkcc was employed as the outgroup in the analysis. ^*^partial sequence.

The coding cDNA sequence of *nkcc* obtained from the gills of *H. signifer* contained 3141 bp (Genbank accession number KF724947), which encoded 1047 amino acids with an estimated molecular mass of 115.6 kDa (Figure 1). It consisted of a N-terminal sequence of around 115 amino acids followed by 12 TMs and a cytosolic C-terminal sequence (Figure 1). Three potential phosphorylation sites and two *N*-glycosylation sites were identified (Figure 1). An alignment of Nkcc of *H. signifer* with those of human, frog, shark and two fishes (seabass and tilapia) revealed that the N-terminus was the least conserved. Notably, both Ste20-related proline-alanine-rich kinase (SPAK) and oxidation stress response kinase 1 (OSR1) interaction sites were absent in the N-terminus of the Nkcc from the gills of *H. signifer* (Figure 1). The Nkcc of *H. signifer* shared the greatest percentage similarity with Nkcc1 of *S. acanthias* (83%), and it had greater percentage similarities with Nkcc of other fishes (52–83%) than to those of amphibians (70%) and mammals (56–70%). Indeed, a phylogenetic analysis confirmed that Nkcc of *H. signifer* was closer to *S. acanthias* and mammal Nkcc1 than to teleost Nkcc1 or Nkcc2 (Figure 2). Hence, the Nkcc obtained from the gills of *H. signifer* was identified as Nkcc1.

### Changes in branchial mRNA expression of *nkcc1*, in response to brackish water acclimation

**Figure 3:**
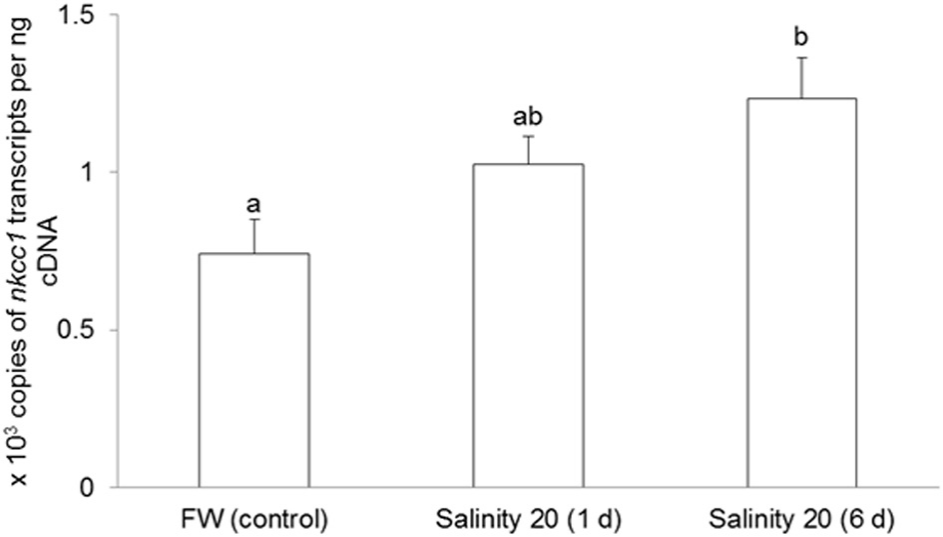
**Effects of acclimation to brackish water on *Na^+^:K^+^:2Cl*^−^*cotransporter 1* (*nkcc1*) mRNA expression in the gills of *Himantura signifer*.** Absolute quantification (copies of transcript per ng of cDNA) of mRNA expression of *nkcc1* in gills of *H. signifer* kept in fresh water (FW; control) or exposed to brackish water (salinity 20) for 1 or 6 d. Results represent means + s.e.m. (*N* = 5). Means not sharing the same letter are significantly different (*P* < 0.05).

The mRNA expression of *nkcc1* in the gills of *H. signifer* exposed to brackish water for 1 d remained unchanged as compared to the freshwater control, but it increased significantly (1.67-fold) after 6 d of exposure to brackish water (Figure 3).

### Nucleotide sequences of *nka*α*1*, *nka*α*2* and *nka*α*3*, and their deduced amino acid sequences

**Figure 4:**
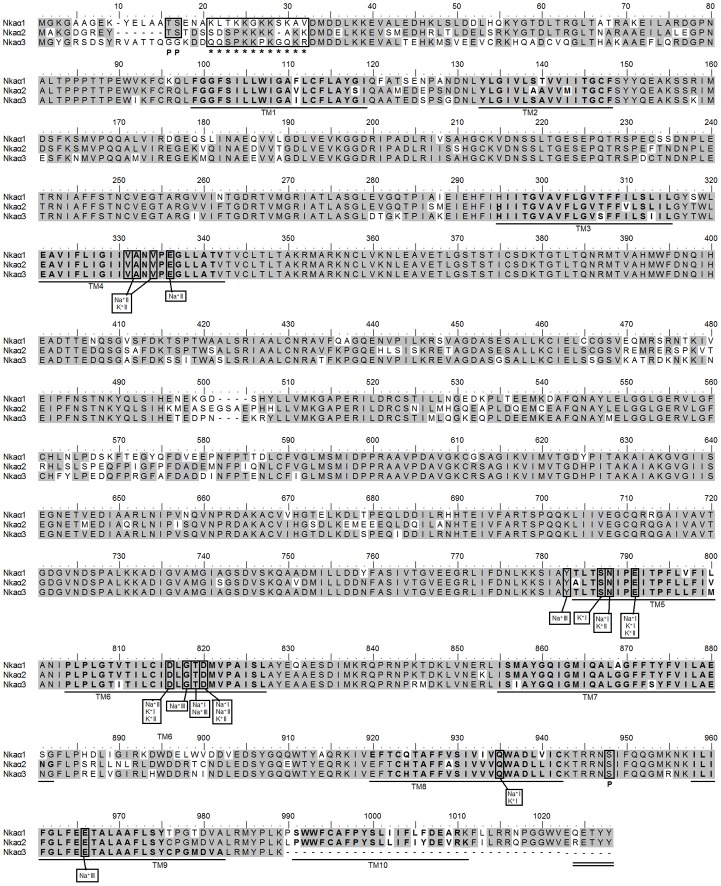
**Molecular characterization of Na^+^/K^+^-ATPase (Nka) α1, Nkaα2, and Nkaα3 from the gills of *Himantura signifer*.** A multiple amino acid sequence alignment of Nkaα1, Nkaα2, and Nkaα3 from the gills of *H. signifer*. Identical amino acid residues are indicated by shaded residues. The ten predicted transmembrane regions (TM1-TM10) are underlined and in bold. Vertical boxes represent coordinating residues for Na^+^ or K^+^ binding. “P” denotes phosphorylation sites and asterisks indicate the lysine-rich region. The conserved region containing the KETYY sequence motif is double underlined.

Three coding cDNA sequences of *nka*α*1* (GenBank accession number KF724944), *nka*α*2* (KF724945) and *nka*α*3* (KF724946) were cloned from the gills of *H. signifer*. The coding sequence of *nka*α*1* contained 3072 bp, which encoded 1024 amino acids with an estimated molecular mass of 112.4 kDa (Figure 4). For *nka*α*2*, the coding sequence contained 3066 bp, encoding 1022 amino acids with an estimated molecular mass of 112.7 kDa (Figure 4). In comparison, the cDNA coding sequence of *nka*α*3* was slightly shorter and contained 2961 bp, encoding 987 amino acids with an estimated molecular mass of 108.3 kDa (Figure 4). A hydropathy analysis revealed that NKAα1, NKAα2 and NKAα3 comprised 10 transmembrane domains (Figure 4). Nkaα1, Nkaα2 and Nkaα3 from gills of *H. signifer* were found to contain conserved regions and residues including a lysine-rich region near the N-terminus and a phosphorylation site (Ser948) that could serve as a target for protein kinase A (Figure 4). While potential targets for regulatory phosphorylation (Thr16 and Ser17) by protein kinase C were present in both Nkaα1 and Nkaα2, they were absent from Nkaα3 (Figure 4).

**Figure 5:**
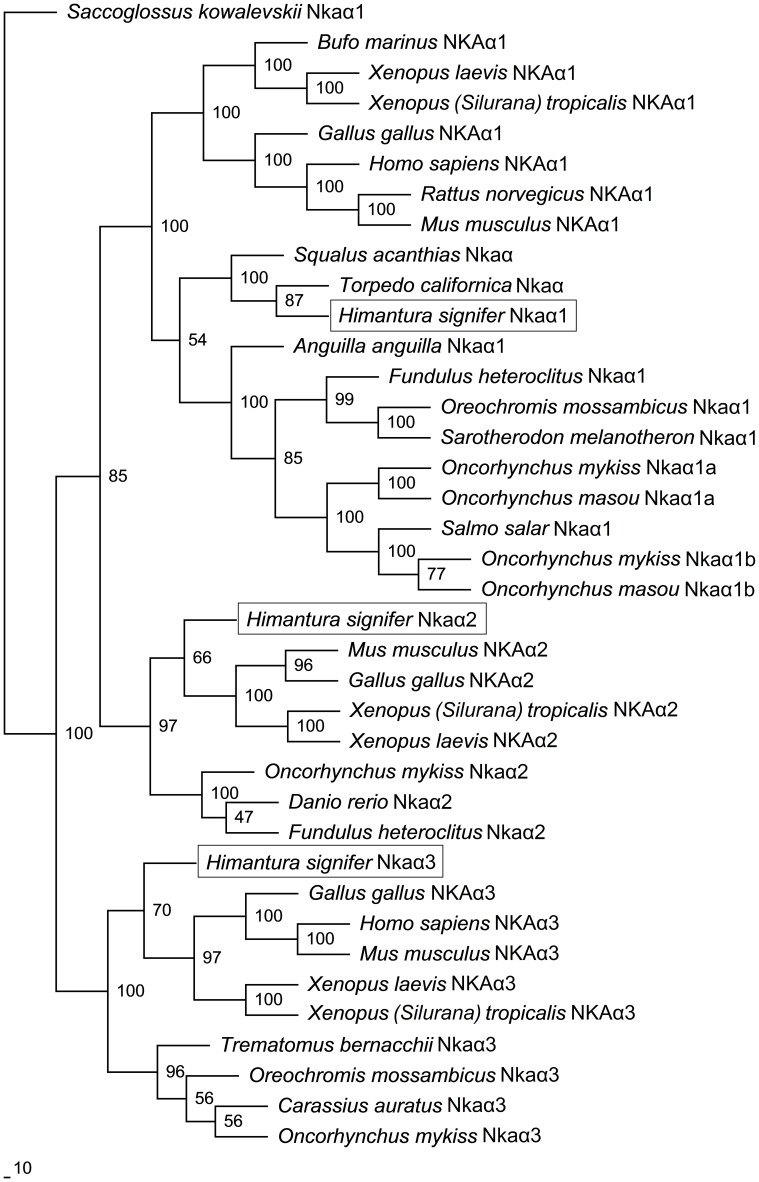
**Phylogenetic analysis of Na^+^/K^+^-ATPase (Nka) α1, Nkaα2, and Nkaα3 from gills of *Himantura signifer*.** A phylogenetic tree illustrating the relationships between Nkaα1, Nkaα2, and Nkaα3 of *H. signifer* and other NKAα/Nkaα isoforms of selected vertebrates.

A comparison of Nkaα1, Nkaα2 and Nkaα3 of *H. signifer* with Nka α-subunits of teleosts and NKA α-subunits from other animals showed that they shared the highest amino acid sequence identity with teleosts Nkaα1 (84–85%), Nkaα2 (84%) and Nkaα3 (84–88%), respectively. Indeed, a phylogenetic analysis of the amino acid sequences confirmed that the three Nkaα isoforms cloned from the gills of *H. signifer* were Nkaα1, Nkaα2 and Nkaα3 (Figure 5). Nkaα1 from *H. signifer* was grouped with those from *S. acanthias* and *Torpedo californica*, and was closer to teleosts than to tetrapods. By contrast, Nkaα2 and Nkaα3 were apparently closer to tetrapods than to teleosts (Figure 5).

**Figure 6:**
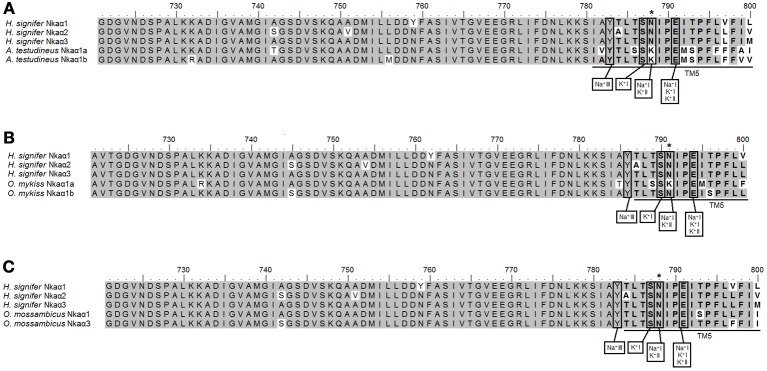
**Analysis of Na^+^ and K^+^ binding sites of Na^+^/K^+^-ATPase (Nka) α1, Nkaα2 and Nkaα3 from the gills of *Himantura signifer*.** A multiple amino acid sequence alignment of a region of Nkaα1, Nkaα2, and Nkaα3 from the gills of *H. signifer*, with **(A)** Nkaα1a (JN180940) and Nkaα1b (JN180941) from the gills of *Anabas testudineus*, **(B)** Nkaα1a (NP_001117933) and Nkaα1b (NP_001117932.1) from the gills of *Oncoryhnchus mykiss*, and **(C)** Nkaα1 (AAD11455.2) and Nkaα3 (Genbank: AAF75108.) from the gills of *Oreochromis mossambicus*. Identical amino acid residues are indicated by shaded residues. Vertical boxes represent coordinating residues for Na^+^ or K^+^ binding. For **(A)**, an asterisk indicates the amino acid residue that is different from Nkaα1a and Nkaα1b. For **(B)**, an asterisk indicates the amino acid residue that is similar to Nkaα1b but different from Nkaα1a. For (C), an asterisk indicates the amino acid residue that is similar to Nkaα1 and Nkaα3.

A detailed analysis of the amino acid residues constituting the K^+^ binding sites of Nkaα1, Nkaα2 and Nkaα3 from the gills of *H. signifer* revealed that there was no replacement of Asn by Lys in position 788/791 (according to the alignment reported in this manuscript) as shown in Nkaα1a and Nkaα1b of climbing perch (*A. testudineus*; Figure 6A) and Nkaα1a of trout (*O. mykis*s; Figure 6B). In contrast, the sites were identical to those of tilapia (*O. mossambicus*; Figure 6C).

### Changes in branchial mRNA expression of *nka*α*1*, *nka*α*2* and *nka*α*3*, in response to brackish water acclimation

**Figure 7:**
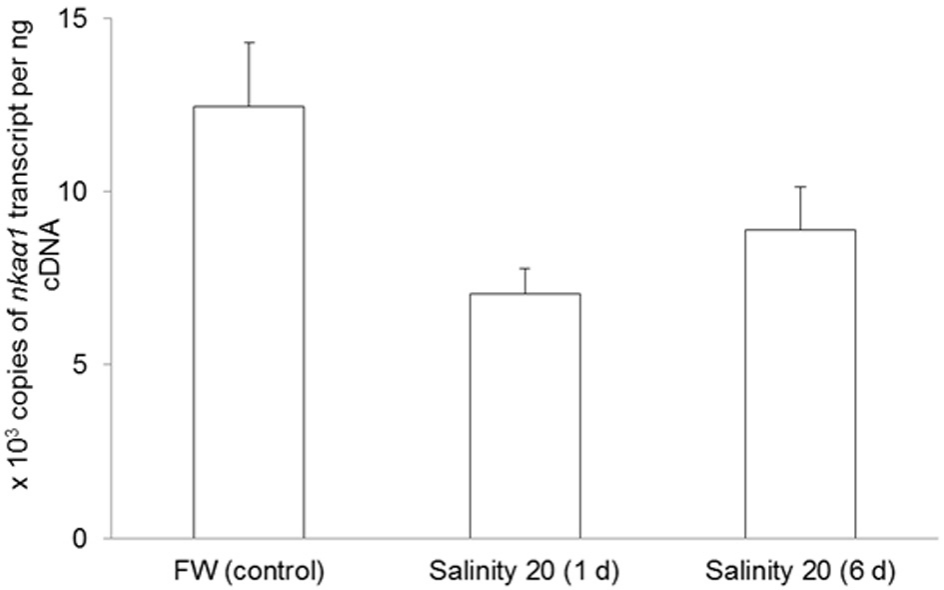
**Absolute quantification of mRNA expression of *Na^+^/K^+^-ATPase α1* (*nka*α*1*) in the gills of *Himantura signifer*.** mRNA expression (copies of transcript per ng cDNA; *N* = 4) of *nka*α*1* in gills of *H. signifer* kept in fresh water (FW, control) or exposed to brackish water (salinity 20) for 1 or 6 d after a progressive increase in salinity. Results represent means ± s.e.m.

**Figure 8:**
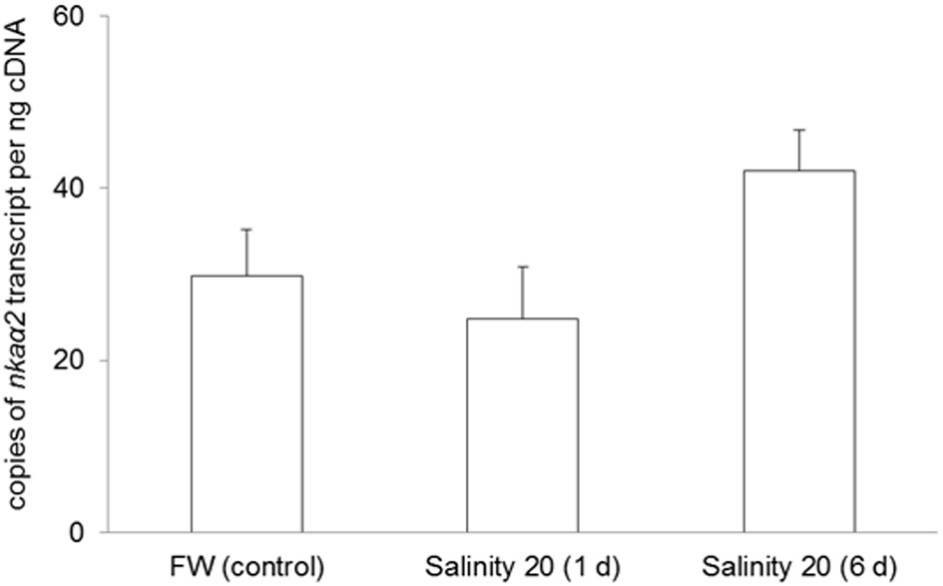
**Absolute quantification of mRNA expression of *Na^+^/K^+^-ATPase α2* (*nka*α*2*) in the gills of *Himantura signifer*.** mRNA expression (copies of transcript per ng cDNA; *N* = 4) of *nka*α*2* in gills of *H. signifer* kept in freshwater (FW, control) or exposed to brackish water (salinity 20) for 1 or 6 d after a progressive increase in salinity. Results represent means ± s.e.m.

**Figure 9:**
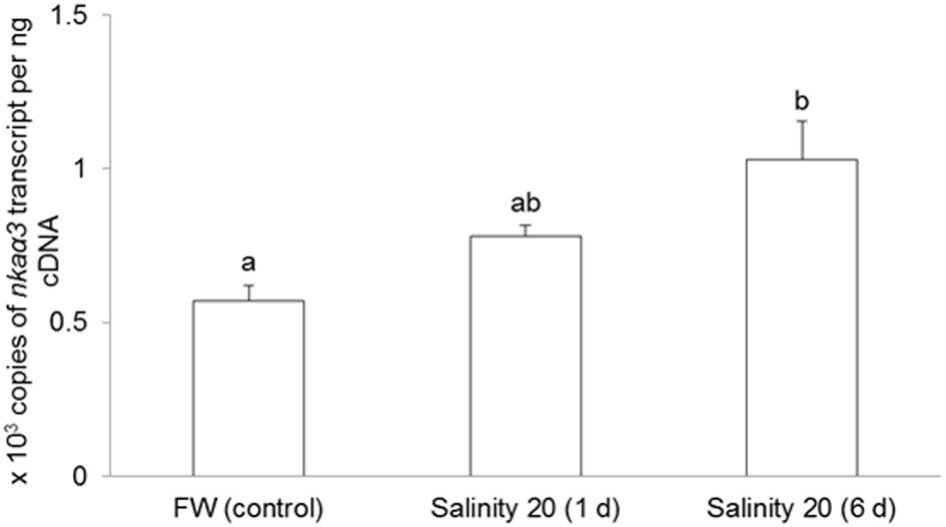
**Absolute quantification of mRNA expression of *Na^+^/K^+^-ATPase α3* (*nka*α*3*) in the gills of *Himantura signifer*.** mRNA expression (copies of transcript per ng cDNA; *N* = 4) of *nka*α*3* in gills of *H. signifer* kept in fresh water (FW, control) or exposed to brackish water (salinity 20) for 1 or 6 d after a progressive increase in salinity. Results represent means ± s.e.m. Means not sharing the same letter are significantly different (*p* < 0.05).

No changes were observed in the mRNA expression of *nka*α*1* (Figure 7) and *nka*α*2* (Figure 8) in the gills of *H. signifer* after 1 or 6 days of exposure to brackish water. By contrast, the mRNA expression of *nka*α*3* in gills of *H. signifer* increased significantly (1.49-fold) in fish exposed to brackish water for 6 days (Figure 9).

### Western blotting of Nkaα

**Figure 10:**
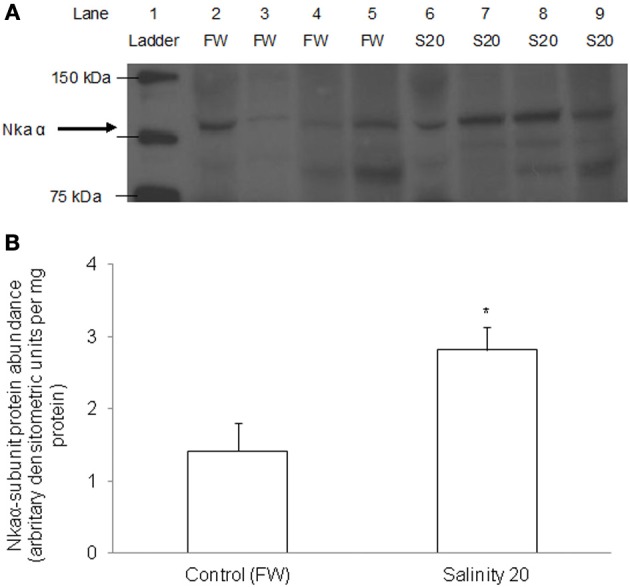
**Protein abundance of Nkaα in the gills of *Himantura signifer*.** Protein abundance of Nkaα in the gills of *H. signifer* kept in fresh water (FW; control), or after 6 d of exposure to salinity 20 (S20). **(A)** Immunoblot of Nka α. **(B)** The intensity of the Nka α protein band per mg protein. Results represent means ± s.e.m. (*N* = 4). ^*^Significantly different from the FW control (*P* < 0.05).

There was a significant increase in the protein abundance of Nkaα in the gills of *H. signifer* acclimated to brackish water for 6 d as compared with the freshwater control (Figure 10).

### Immunofluorescence microscopy

**Figure 11:**
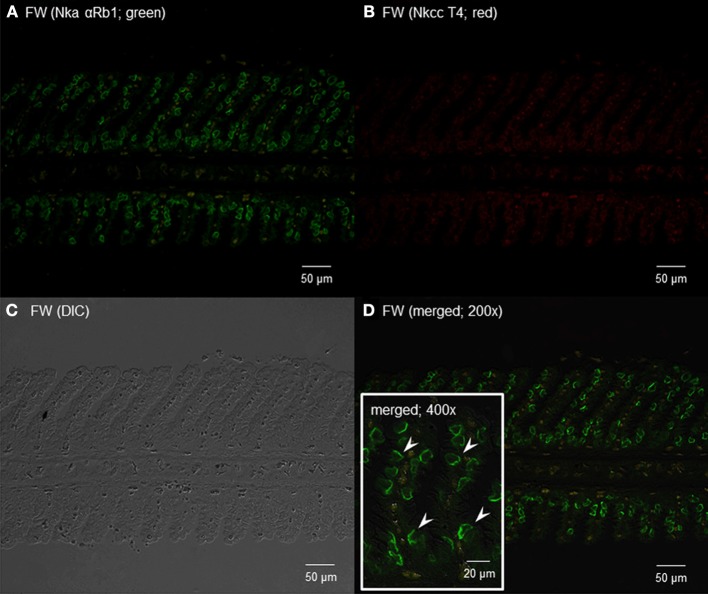
**Immunofluorescent localization of Na^+^/K^+^-ATPase α-subunit (Nkaα) and Na^+^:K^+^:2Cl^−^ cotransporter (Nkcc) in the gills of *Himantura signifer* kept in fresh water (FW; control).** Immunofluorescence using **(A)** anti-NKA αRb1 antibody (green) or **(B)** anti-NKCC T4 antibody (red). The differential interference contrast image (DIC) is shown in **(C)**. All channels (green and red) are merged and overlaid with DIC in **(D)**. Arrowheads in the inset of **(D)** denote the staining of Nkaα in presumably a type of FW ionocytes. Magnification: 200× for **(A–C)** and **(D)**, or 400× for inset of **(D)**. Reproducible results were obtained from three individuals.

**Figure 12:**
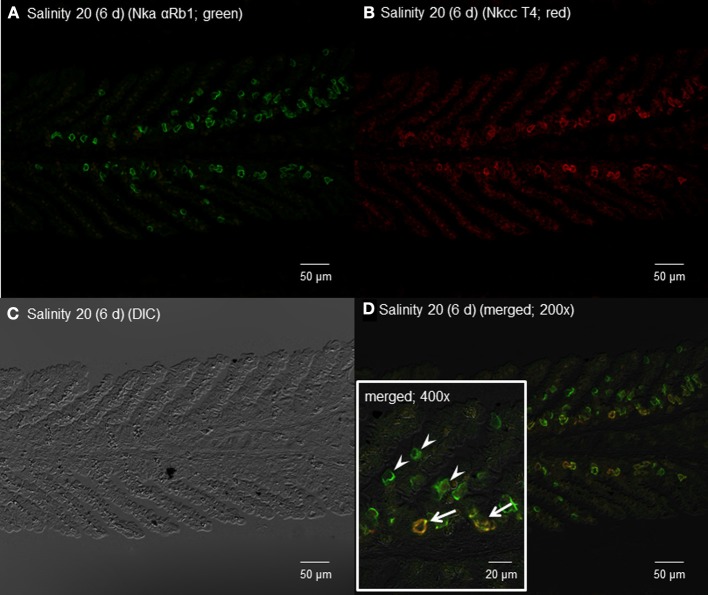
**Immunofluorescent localization of Na^+^/K^+^-ATPase α-subunit (Nkaα) and Na^+^:K^+^:2Cl^−^ cotransporter (Nkcc) in the gills of *Himantura signifer* exposed to brackish water (BW; salinity 20) for 6 d after a progressive increase in salinity.** Immunofluorescence using **(A)** anti-NKA αRb1 antibody (green) or **(B)** anti-NKCC T4 antibody (red). The differential interference contrast image (DIC) is shown in **(C)**. All channels (green and red) are merged and overlaid with DIC in **(D)**, whereby integration of red and green channels resulted in a yellow-orange coloration. In the inset of **(D)**, arrows indicate the staining of Nka and Nkcc in a type of BW ionocyte, while arrowheads denote the staining of only Nka in another type of ionocyte. Magnification: 200× for **(A–D)**, or 400× for inset of **(D)**. Reproducible results were obtained from three individuals.

Using the rabbit anti-NKA polyclonal antibody, αRb1NKA, which reacted comprehensively with fish Nka α-subunit isoforms, immunoreactivity was demonstrated in the gills of *H. signifer* (Figures 11, 12). The αRb1NKA-labelled ionocytes were distributed throughout the secondary lamellae of fish in fresh water (Figures 11A,D). However, using the T4 anti-NKCC antibody, only weak auto-fluorescence of erythrocytes (as reflected in the negative control) was observed in the gills of *H. signifer* kept in fresh water (Figure 11B). According to Lytle et al. (1995) and Hiroi et al. (2008), the T4 anti-NKCC antibody can react with NKCC1, NKCC2 and Na^+^:Cl^−^ cotransporter (NCC), but *nkcc2*/Nkcc2 is known to be expressed mainly in fish intestine (Kato et al., 2011) and kidney (Kim et al., 2013). Although NCC is known to be involved in ion absorption in freshwater-type ionocytes in freshwater teleosts, our results indicated that Ncc or Nkcc1 were undetectable in the gills of freshwater *H. signifer*.

By contrast, 6 days of exposure to brackish water led to the appearance of some ionocytes with strong immunofluorescence with αRb1NKA near the base of the secondary lamellae in the gills of *H. signifer* (Figures 12A,D). In addition, some cells near the base of the secondary lamellae were immunoreactive to the T4 anti-NKCC antibody (Figure 12B). Nka and Nkcc were apparently colocalized to the basolateral membrane of some of these ionocytes (Figure 12D, D'), while there were other ionocytes, which expressed only Nka and not Nkcc, along the secondary lamellae (Figure 12D).

## Discussion

### Expression of *nkcc1* in gills of *H. signifer* in fresh water and its upregulation during brackish water acclimation

The electroneutral NKCC belongs to the SLC12A family and is an integral membrane protein responsible for transporting one Na^+^, one K^+^, and two Cl^−^ ions simultaneously from the external to the internal side of epithelial cells (Russell, 2000; Gamba, 2005). NKCC is involved in ion transport across secretory and absorptive epithelia (Haas and Forbush, 1998). NKCC has two isoforms, NKCC1 and NKCC2 (Haas and Forbush, 2000). In mammals, NKCC1 is present in many cell types (Yan et al., 2001; Wang et al., 2003), while NKCC2 is localized exclusively to the kidney (Ecelbarger et al., 1996). NKCC1 is inactive in the basolateral membranes of secretory cells but can be activated through phosphorylation as a result of cell shrinkage or the presence of secretagogues (Lytle and Forbush, 1992; Haas et al., 1995). Based on percentage identity and phylogenetic analysis, the Nkcc obtained from the gills of *H. signifer* was Nkcc1.

Both marine teleosts and marine elasmobranchs are confronted with the influx of NaCl, but marine teleosts lose water while marine elasmobranchs have to cope with a modest water influx. Thus, marine teleosts imbibe seawater and excrete excess Na^+^ and Cl^−^ via the gills to prevent dehydration. However, marine elasmobranchs do not drink seawater and eliminate excess Na^+^ and Cl^−^ through the rectal gland. Consequently, gills of marine elasmobranchs play only a very minor role in salt balance. Since elasmobranch gills are not known to express Nkcc1 and to excrete salt, the expression of *nkcc1* in the gills of *H. signifer* is a novel discovery.

Studies on shark rectal gland have shown an increase in phosphorylation of serine and threonine of Nkcc1 in response to forskolin (a cyclic AMP agonist known to regulate NKCC) or hypertonic stress, and many of the phosphoregulatory sites are found in the N terminus (Lytle and Forbush, 1992; Kurihara et al., 1999). Darman and Forbush (2002) demonstrated that at least three sites in NKCC are phosphorylated upon stimulation, and our results indicated that these three phosphorylation sites (Thr229, Thr234, and Thr247, based on the alignment reported in this manuscript) were conserved in the Nkcc1 of *H. signifer*. All NKCC are glycoproteins (Lytle et al., 1992; Darman and Forbush, 2002) which possess sites for *N*-linked glycosylation within a large hydrophilic loop between putative TM7 and TM8 (Payne and Forbush, 1995), and the consensus sites for *N*-linked glycosylation had been identified in the Nkcc1 of *H. signifer*. The presence of the phosphorylation and glycosylation sites in the Nkcc1 of *H. signifer* indicates that it can be regulated through post-translational modification in response to changes in environmental conditions. Piechotta et al. (2002, 2003) identified a SPAK and oxidation stress response kinase 1 (OSR1) interaction site in the N-terminus of Nkcc, and suggested that sea bass Nkcc1 may be activated by stress kinases in response to salinity changes. However, both SPAK and OSR1 interaction sites are absent from the N-terminus of Nkcc1 of *H. signifer*. This suggests that the activation of Nkcc1 in the gills of *H. signifer* through phosphorylation and/or glycosylation by osmotic and/or oxidative stresses would not be effective.

More importantly, we report for the first time an increase in the expression of mRNA expression of *nkcc1* in the gills of the freshwater *H. signifer* acclimated to brackish water, alluding to its involvement in the excretion of excess Na^+^ and Cl^−^. In comparison with other euryhaline freshwater teleosts which can endure seawater acclimation, the increase in *nkcc1* mRNA expression in the gills of *H. signifer* was moderate (~2-fold). For instance, there was >11-fold increase in Nkcc1 in the gills of the euryhaline freshwater climbing perch, *A. testudineus*, after 6 days of acclimation to seawater (Loong et al., 2012). Taken together, our results indicate that *H. signifer* has a limited capacity for branchial osmoregulatory acclimation, which could have contributed in part to its inability to survive in seawater or to return to a completely marine environment.

### Expression of three *nka*α isoforms in gills of *H. signifer* in fresh water and the upregultion of *nka*α*3* mRNA expression and nkaα protein abundance during brackish water acclimation

During salt excretion, the increased operation of Nkcc1, which cotransports Na^+^, K^+^, and Cl^−^ through the basolateral membrane down the electrochemical gradient of Na^+^ provided by Nka, would lead to an increase in the influx of Na^+^ into the ionocyte. In marine teleosts that have a transepithelial electrical potential of 25–35 mV (blood side positive; Wright, 1991; Evans et al., 1999; Marshall, 2002) intracellular Na^+^ homeostasis is maintained by Nka, which actively transport Na^+^ back to the blood so that Na^+^ can be excreted through the paracellular route. Hence, it was important to examine whether brackish water acclimation would led to an up-regulation of Nka activity to accommodate for the increased Nkcc1 activity in the gills of *H. signifer*.

Nka is a key enzyme involved in the active transport of Na^+^ and Cl^−^ in osmoregulatory tissues, including gills, kidney, and intestine of teleosts and also the rectal gland of elasmobranchs. In marine teleosts it is the active and rate limiting step in the excretion of excess Na^+^ and Cl^−^ by ionocytes in the branchial epithelia (Marshall and Bryson, 1998) and is important in the osmoregulatory plasticity of euryhaline fish. However, no information on isoforms of *nka*α is available for gills of elasmobranchs, although the complete cDNA sequence of a single isoform of *nka α*-subunit has been determined from the electric organ of the torpedo ray, *T. californica* since 1995 (Kawakami et al., 1995). Here, we demonstrate for the first time the expression of three *nka*α isoforms in the gills of *H. signifer*.

Three Na^+^ and two K^+^ binding sites are known to be present in the NKA α-subunit (Ogawa and Toyoshima, 2002; Li et al., 2005). The coordinating residues present in the binding sites are arranged within the transmembrane domains such that the release of one type of cation coordinates with the binding of the other. Based on the homology modeling of human NKAα (Ogawa and Toyoshima, 2002), these coordinating residues are conserved in Nkaα1, Nkaα2, and Nkaα3 from the gills of *H. signifer*. It has been established that Na^+^ and K^+^ are occluded within NKA during each turnover of the pump and this occlusion requires conformational changes in the enzyme (Glynn et al., 1984). Proteolytic cleavage at a lysine-rich region near the N-terminus alters the equilibrium between the E1 and E2 conformations (Jørgensen and Karlish, 1980). Hence, this conformational shift could involve the movement of the lysine-rich sequence, which could serve as a movable ion-selective gate, controlling the passage of Na^+^ and K^+^ during certain stages of the transport process (Shull et al., 1986). Indeed, the highly conserved lysine-rich sequence is present in Nkaα1, Nkaα2, and Nkaα3 from the gills of *H. signifer*, indicating that they might share close structural-functional relationships with those of other animal species.

Morth et al. (2007) reported that there was a 26-fold reduction in Na^+^ affinity when five amino acid residues (KETYY) were deleted from the C-terminal of NKA. In *H. signifer*, the KETYY motif was present in both Nkaα1 and Nkaα2, but it is missing from Nkaα3, the C terminus of which had 39 less amino acids. This would imply that Nkaα3 might have lower Na^+^ affinity than Nkaα1 and Nkaα2 in the gills of *H. signifer*. In addition, the lysine residue was replaced by glutamine and arginine in Nkaα1 and Nkaα2, respectively. This provides further indication to the differences in the Na^+^ affinity of the three forms Nkaα1, Nkaα2, and Nkaα3. Mutation studies have shown that asparagine 786 is critical for both Na^+^ and K^+^ binding (Pedersen et al., 1998). However, no replacement of asparagine (Asparagine-788/791 according to the alignment reported in this manuscript) was found in Nkaα1, Nkaα2, and Nkaα3 of *H. signifer* and they are instead similar to Nkaα1b of *O. mykiss* and Nkaα1 and Nkaα3 of *O. mossambicus*.

Our results suggest that Nka could be regulated by phosphorylation/ dephosphorylation in the gills of *H. signifer*. Both cAMP-dependent protein kinase A and protein kinase C are known to be involved in the phosphorylation of the NKAα (Aperia et al., 1994) although the functional effects of protein kinases remain controversial (Feschenko and Sweadner, 1997). One possible site of cAMP-dependent protein kinase A phosphorylation is the serine residue at position 948 (according to the alignment reported in this manuscript) of the NKAα-subunit from the kidneys of the rat and *B. marinus* (Beguin et al., 1994; Feschenko and Sweadner, 1995). This site is apparently conserved in the three Nkaα-subunit isoforms from the gills of *H. signifer*. Beguin et al. (1994) identified Threonine-10 and Serine-11 as cAMP-dependent protein kinase C phosphorylation sites in the NKA of *B. marinus* by site-directed mutagenesis. This corresponds to Threonine-16 and Serine-17 in Nkaα1 of *H. signifer* but are absent in Nkaα3. Feschenko and Sweadner (1995) identified two cAMP-dependent protein kinase C phosphorylation sites, Serine-11 and Serine-18, with different phosphorylatability in rat kidney NKAα1. In comparison, only one cAMP-dependent protein kinase C phosphorylation site (serine 17) was found in Nkaα1 and Nkaα2 from the gills of *H. signifer*, but both serine residues were absent in Nkaα3. Overall, it would appear that Nkaα3 could be regulated to a lesser extent by post-translational modification than Nkaα1 and Nkaα2 in the gills of *H. signifer* in response to environmental changes. Conversely, it would imply that Nkaα3 could be subjected to transcriptional regulation more than Nkaα1 and Nkaα2.

Gills of elasmobranchs acquired an iono-regulatory function only after they invaded the freshwater habitat (Evans, 1984). The branchial Nka activities of marine elasmobranchs are 1/10 those of marine teleosts (Jampol and Epstein, 1970). Furthermore, the basolateral membrane of ionocytes of marine elasmobranchs has much less infolding (Wright, 1973), indicating that they are not involved in salt excretion. In fresh water, the gills of elasmobranchs function more like those of freshwater teleosts, and uptake of Na^+^ and Cl^−^ become important as demonstrated in the stenohaline freshwater *Potamotrygon* (Pang et al., 1977; Wood et al., 2002). For the euryhaline marine *D. sabina*, the activity and protein abundance of Nkaα in the gills of fish acclimated to fresh water is higher than those of fish acclimated to seawater (Piermarini and Evans, 2000). By contrast, we demonstrate for the first time that acclimation of *H. signifer* from fresh water to brackish water led to significant increases in the mRNA expression of *nka*α*3* and the overall protein abundance of Nkaα in its gills. It has been reported that seawater acclimation in rainbow trout (*O. mykiss*), Atlantic salmon (*S. salar*) and arctic char (*Salvelinus alpinus*) involves the differential regulation of two gill *nka*α isoforms, *nka*α*1a* and *nka*α*1b* (Richards et al., 2003; Bystriansky et al., 2006). When salmonids are exposed to seawater, the rise in Nka activity is preceded by increased expression of *nka*α*1b*, which suggests that this isoform is important for successful acclimation to seawater. On the other hand, the rapid decrease in *nka*α*1a* expression upon seawater exposure suggests that Nkaα1a could be involved in ion uptake (Bystriansky et al., 2007). McCormick et al. (2009) developed and validated rabbit polyclonal antibodies specific to the Nkaα1a and Nkaα1b isoforms of Atlantic salmon (*S. salar*), and used Western blotting and immunohistochemistry to characterize their size, abundance and localization. Their results indicated that there were a freshwater Nkaα isoform and a seawater Nkaα isoform, which were present in distinct ionocytes, in the gills of *S. salar*. Furthermore, McCormick et al. (2013) reported that Nkaα isoforms were differentially regulated in *S. salar* during smolt development and seawater exposure. Similarly, Ip et al. (2012) and Ching et al. (2013) identified *nka*α*1a*/Nkaα1a and *nka*α*1b*/Nkaα1b as the freshwater- and seawater-isoforms, respectively, in the gills of the freshwater *A. testudineus* which also has distinct freshwater-type and seawater-type ionocytes. For *H. signifer*, ion absorption in fresh water appeared to depend on *nka*α*1* which was the major isoform (~10 × 10^3^ copies of transcripts ng^−1^ cDNA), while survival in brackish water depended on *nka*α*3* (0.1 - 0.8 × 10^3^ copies of transcripts ng^−1^ cDNA), the upregulation of which could have contributed in part to a significant increase in the Nkaα protein abundance. When taken together with results on *nkcc1*/Nkcc1, it can be concluded that the gills of *H. signifer* probably have an iono-regulatory role in salt excretion during brackish water acclimation.

### Ionocytes in gills of *H. signifer*

The current model of elasmobranch gills prescribes two types of ionocytes, both of which are present in freshwater and marine species, but they lack Nkcc1 (Ballantyne and Robinson, 2010). One type of ionocyte has a basolateral vH^+^-ATPase and an apical HCO^−^_3_/Cl^−^ exchanger (Piermarini et al., 2002). The vH^+^-ATPase has been localized to the basolateral membrane of ionocytes in gills of both marine and freshwater elasmobranchs (Piermarini and Evans, 2001), and the number of ionocytes with vH^+^-ATPase increases in fresh water (Wood et al., 2003). Hence, in fresh water, this type of ionocyte probably functions in HCO^−^_3_ excretion and Cl^−^ uptake (Wood et al., 2003; Evans et al., 2004). The other type of ionocyte has a basolateral Nka and an apical Na^+^/H^+^ exchanger (Choe et al., 2005, 2007). The apical Na^+^/H^+^ exchanger plays a role in acid–base regulation in general (Choe et al., 2007), and in Na^+^ uptake during fresh water acclimation. Furthermore, an H^+^/K^+^-ATPase has been demonstrated in the gills of *D. sabina* with the expression increasing in fresh water presumably to facilitate K^+^ uptake (Choe et al., 2004). In fresh water, increases in expression of HCO^−^_3_/Cl^−^ exchanger (Piermarini et al., 2002), H^+^/K^+^-ATPase (Choe et al., 2004) and Nka (Piermarini and Evans, 2000) occur to enhance ion absorption. Our results demonstrate for the first time the existence of a novel type of ionocyte, which co-expressed Nkcc1 and Nkaα along the basolateral membrane, at the base of the secondary lamellae in the gills of *H. signifer* acclimated to brackish water. This brackish water type of ionocyte was not found in gills of *H. signifer* kept in fresh water. Instead, the gills of freshwater *H. signifer* possessed another type of Nka-immunoreactive ionocyte without Nkcc1 distributed along the secondary lamellae. Hence, it is logical to deduce that there could be a change in the function of the gills of *H. signifer* from salt absorption to salt excretion during acclimation to brackish water.

Incidentally, four distinct types of ionocytes have been identified in the yolk-sac membrane of tilapia embryos (Hiroi et al., 2008): type I with only basolateral Nka, type II with basolateral Nka and apical Na^+^:Cl^−^ cotransporter, type III with basolateral Nka and basolateral Nkcc1a, and occasionally with apical Na^+^/H^+^ exchanger 3 (Nhe3), and type IV with basolateral Nka, basolateral Nkcc1a and apical Cftr (Hiroi and McCormick, 2012). Based on the types of transporter present, it is apparent that type IV ionocyte is involved in ion secretion in seawater. By contrast, type III ionocyte functions to absorb ions and/or regulate acid/base balance due to the lack of Cftr and presence of Nhe3 at the apical membrane. Indeed, type III ionocyte rarely appears in seawater, increases rapidly in number in response to transfer from seawater to fresh water, and disappears after transfer from fresh water to seawater. It is possible that type III ionocyte can be rapidly transform to type IV ionocyte by the expression of Cftr at the apical membrane during seawater acclimation (Hiroi and McCormick, 2012). Similarly, four distinct types of ionocytes have been identified in the skin and gills of zebrafish (Hwang et al., 2011), and there are some similarities between the characteristics of tilapia type III ionocyte and those of zebrafish H^+^-ATPase-rich (HR) ionocyte in fresh water. However, HR ionocyte cannot be transformed into type IV ionocyte for ion secretion as zebrafish is stenohaline to fresh water (Hiroi and McCormick, 2012). In contrast with tilapia and zebrafish, the (Nka + Nkcc)-immunoreactive type of ionocyte appeared only in the gills of *H. signifer* exposed to brackish water and not in those kept in fresh water. Therefore, it is highly probable that this brachish water-type ionocyte of *H. signifer* is dissimilar to type III ionocyte of tilapia and HR ionocyte of zebrafish, but whether it is similar to type IV ionocyte of tiplapia is uncertain at present. Efforts should be made in the future to elucidate whether apical Cftr is co-expressed with basolateral Nkcc1 and Nka in the brackish water type of ionocyte, and to determine the transepithelial electric potential, in *H. signifier*. For Na^+^ to be excreted through the paracellular route in the gills of *H. signifier*, as in the case of those marine teleosts which have a transepithelial electrical potential of 25–35 mV (blood side positive; Evans et al., 1999; Marshall, 2002), it is crucial for Cl^−^ that has entered the ionocyte through Nkcc1 to be transported to the external medium through Cftr, contributing to the generation of a transepithelial electrical potential equal to or greater than the Nernst equilibrium potential of Na^+^.

Changes in ionocyte types are critical to branchial osmoregulatory acclimation in euryhaline freshwater teleosts. They can be achieved through the modulation of existing ionocytes, especially when confronted with acute salinity changes (Tsai and Hwang, 1998; Hiroi et al., 1999; Katoh and Kaneko, 2003; Lin and Hwang, 2004; Shen et al., 2011) or through the replacement of existing ionocytes with de novo generated ionocytes (Kammerer and Kültz, 2009; Inokuchi and Kaneko, 2012; Ching et al., 2013). In some cases, the replacement process involves extensive apoptosis in the gills (Ching et al., 2013). Hence, efforts should be made in the future to elucidate whether branchial osmoregulatory acclimation involves the modulation or the replacement of existing ionocytes in the gills of *H. signifer* during brackish water acclimation.

## Conclusion

Despite the rectal gland having a major role in salt excretion (Evans et al., 1979), several experiments have shown that marine elasmobranchs can still osmoregulate after the ligation or removal of their rectal glands (Chan et al., 1967; Haywood, 1975; Evans, 1982; Wilson et al., 2002), alluding to the possibility of an alternative site of ion secretion. Cells similar to ionocytes of teleosts have been described in the branchial epithelium of elasmobranchs (Laurent and Dunel, 1980; Laurent, 1984). However, Wilson et al. (2002) demonstrated that ionocytes in the gills of *S. acanthias* were unresponsive to rectal gland removal with respect to changes in number, fine structure and Nka activity. By contrast, we report for the first time that brackish water acclimation led to increases in mRNA expression of *nkcc1* and *nka*α*3*, and the overall Nkaα protein abundance in the gills of the euryhaline freshwater *H. signifer* which lack a functional rectal gland. In addition, we discovered a novel type of ionocyte which co-expressed Nkcc1 and Nka in the gills of *H. signifer* acclimated to brackish water. Overall, it can be concluded that branchial osmoregulatory acclimation took place in gills of *H. signifer* exposed to brackish water, and the functional role of the rectal gland in salt excretion could have been taken over partially by the gills during brackish water acclimation.

### Conflict of interest statement

The authors declare that the research was conducted in the absence of any commercial or financial relationships that could be construed as a potential conflict of interest.
